# Expression of Cre recombinase in dopaminoceptive neurons

**DOI:** 10.1186/1471-2202-8-4

**Published:** 2007-01-03

**Authors:** Thomas Lemberger, Rosanna Parlato, Donald Dassesse, Magdalena Westphal, Emilio Casanova, Marc Turiault, François Tronche, Serge N Schiffmann, Günther Schütz

**Affiliations:** 1 German Cancer Research Center, 69120 Heidelberg, Germany; 2 Laboratory of Neurophysiology, Université Libre de Bruxelles, 1070 Bruxelles, Belgium; 3 Collège de France, Institut de Biologie, 75231 Paris Cedex 5, France; 4 Molecular Systems Biology, The EMBO Building, D-69117 Heidelberg, Germany; 5 Department of Physiology, Biozentrum, 4056 Basel, Switzerland

## Abstract

**Background:**

Dopamine-activated signaling regulates locomotor and emotional responses and alterations in dopamine-signaling are responsible of several psychomotor disorders. In order to identify specific functions of these pathways, the Cre/loxP system has been used. Here, we describe the generation and the characterization of a transgenic mouse line expressing the Cre recombinase in dopaminoceptive neurons. To this purpose, we used as expression vector a 140 kb yeast artificial chromosome (YAC) containing the dopamine D1 receptor gene (*Drd1a*).

**Results:**

In the chosen line, D1Cre, the spatio-temporal pattern of Cre expression closely recapitulated that of the endogenous *Drd1a *gene, as assessed by immunohistological approaches in embryonic and adult stages. Efficiency of recombination was confirmed by crossing D1Cre with three different loxP lines (*Creb1*^loxP^, *CaMKIV*^loxP ^and *GR*^loxP^) and with the *R26R *reporter. In the three loxP lines studied, recombination was restricted to the area of Cre expression.

**Conclusion:**

In view of the patterns of recombination restricted to the major dopaminoceptive regions as seen in the context of the CREB, CaMKIV and GR mutations, the D1Cre line will be a useful tool to dissect the contributions of specific genes to biological processes involving dopamine signaling.

## Background

Although dopaminergic neurons are few (e.g. 10–20,000 in the rat brain), they regulate a number of physiological, behavioral and cognitive functions, including regulation of locomotor activity, incentive behaviors, short-term and stimulus-dependent memory systems [1-3]. Dysfunction of the dopaminergic system and its targets underlie major human disorders such as Parkinson's disease, Huntington's disease, schizophrenia and addiction to drugs of abuse [4-6]. Five dopamine receptors, termed D1, D2,D3, D4 and D5, encoded by five different genes (*Drd1a*, *Drd2*, *Drd3*, *Drd4 *and *Drd5 *respectively) have been identified [7,8]. The elucidation of the signaling properties of dopaminoceptive neurons is of central importance for the development of therapeutic strategies to treat dysfunctions of the dopaminergic system.

In the past years, use of the Cre/loxP system has enabled generation of cell-specific mutations In the central nervous system, efforts have generally been devoted to the generation of region-specific mutations using promoters to drive the expression of Cre recombinase in specific brain areas [9-11]. In order to target the dopaminoceptive neurons, we have generated a transgenic line that expresses Cre recombinase in dopaminoceptive neurons under the control of the dopamine D1 receptor gene (D1Cre), by using the genomic locus of a dopamine D1 receptor gene cloned in a YAC vector.

Here we examine the cellular expression of Cre recombinase in D1Cre transgenic mice by immunohistochemistry in combination with in situ hybridization, to compare it with the expression of D1 receptor mRNA and other dopaminoceptive molecular markers. The activity of the Cre was confirmed by loss of CREB, CaMKIV and GR proteins in the respective conditional mutants and by induction of beta-galactosidase activity in the *R26R*^D1Cre ^reporter mice.

## Results

A 140 kb yeast artificial chromosome (YAC) containing the entire dopamine D1 receptor gene (*Drd1a*) was isolated by screening a mouse genomic YAC library [12]. The YAC encompasses the entire *Drd1a *gene including 40 kb of upstream and 100 kb of downstream sequences. The coding sequence for the nuclear localized nlsCre [13] was introduced into the *Drd1a *gene by homologous recombination in yeast. To minimize the chances of altering possible regulatory elements, we avoided the use of a heterologous polyadenylation sequence and minimized the deletion of the *Drd1a *coding sequence. Specifically, the homology regions were chosen so that the ATG of the nlsCre would replace the ATG of the *Drd1a *gene and the STOP codon of the nlsCre would interrupt the *Drd1a *open reading frame after its last possible internal ATG.

**Figure 1:**
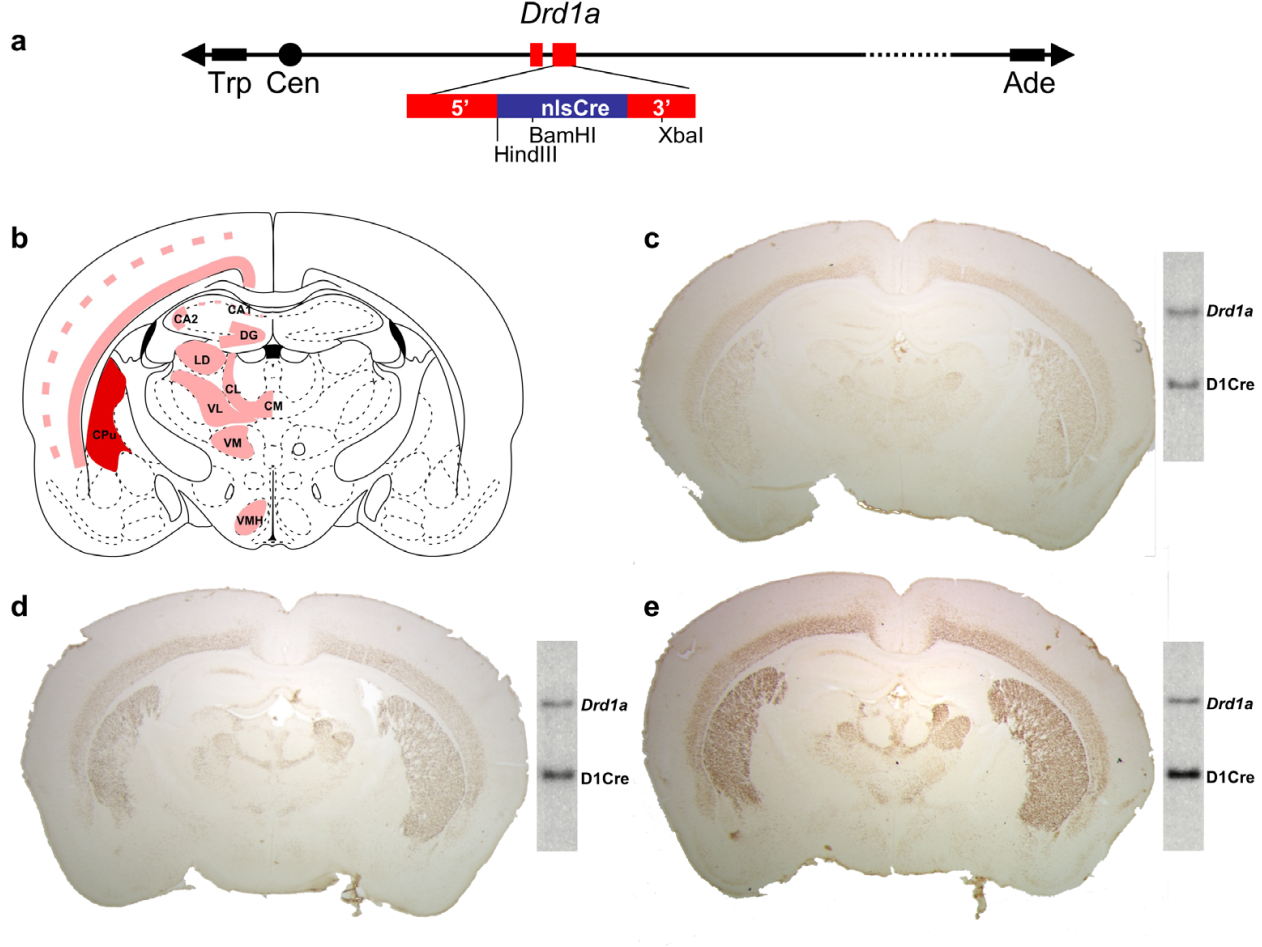
**Position-independent and copy-number dependent expression of the D1Cre YAC transgene**. (**a**) Schematic representation of the modified D1Cre YAC. (**b**) Schematic view of the known distribution of *Drd1a *mRNA [29, 38].(**c-e**) Expression pattern of Cre as revealed by immunohistochemistry in D1Cre transgenic lines. (**c**) Line R, (**d**) line A and (**e**) line S. The accompanying Southern blots show the copy number dependent expression of Cre. The upper band is the endogenous *Drd1a *gene and the lower band is the D1Cre YAC transgene. Abbreviations: CL: centrolateral thalamic nucleus; CM: central medial thalamic nucleus; CPu: caudate putamen; DG: dentate gyrus; LD: laterodorsal thalamic nucleus; VL: ventrolateral thalamic nucleus;VM: ventromedial thalamic nucleus; VMH: ventromedial hypothalamic nucleus. Magnification: (**b-e**) 5×.

**Figure 2:**
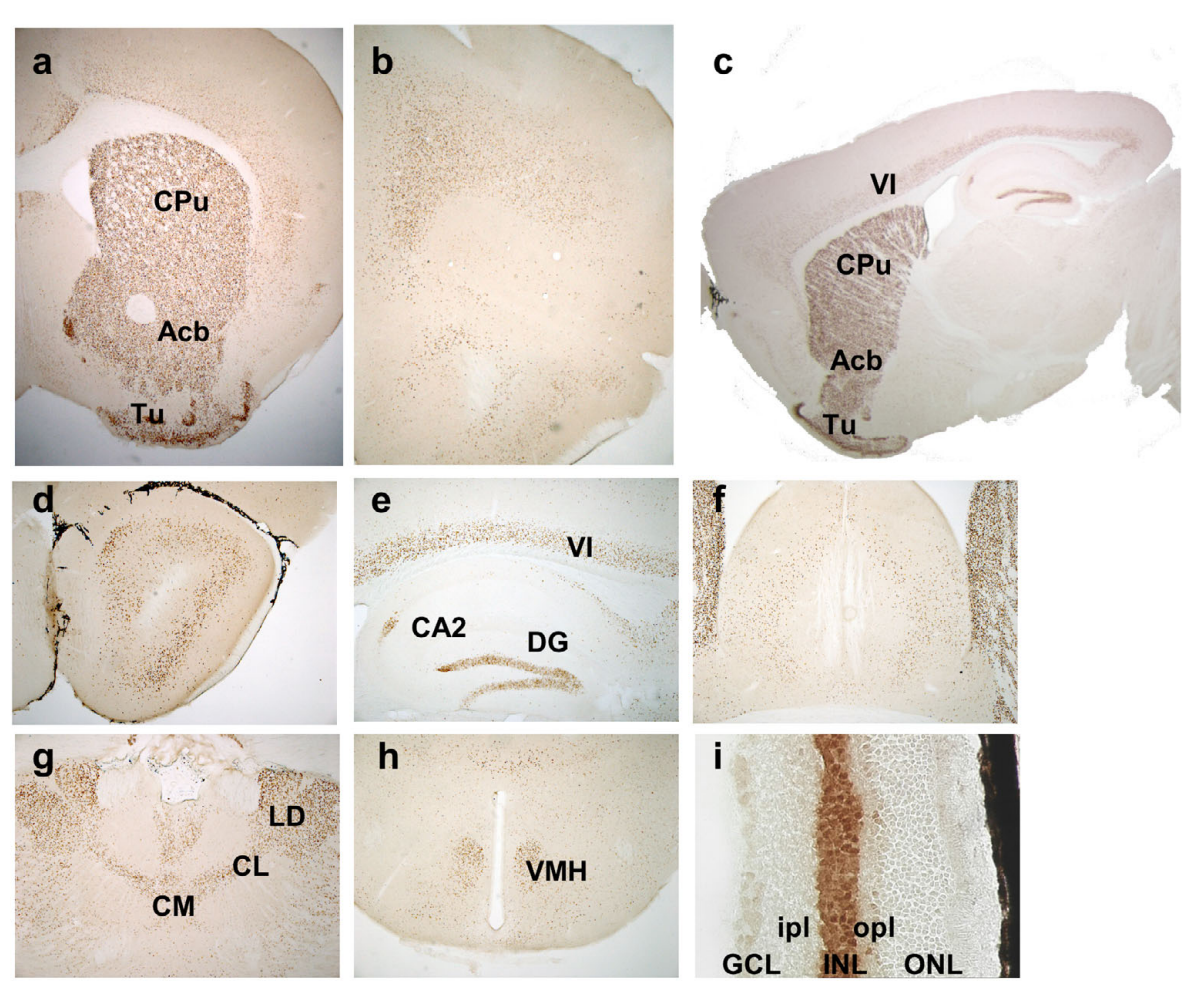
**Detailed pattern of Cre expression**. (**a-i**) Immunohistochemical localization of Cre expression. (**a**) Basal ganglia, (**b**) prefrontal cortex, (**c**) cortex, (**d**) olfactory nucleus, (**e**) hippocampus, (**f**) septum, (**g**) thalamus, (**h**) hypothalamus and (**i**) retina. Abbreviations: Acb: nucleus accumbens; CA2: hippocampus CA2 field; CL: centrolateral thalamic nucleus; CM: central medial thalamic nucleus; CPu: caudate putamen; DG: dentate gyrus; GCL: ganglion cell layer; INL: inner nuclear layer; ipl: inner plexiform layer; LD: laterodorsal thalamic nucleus; ONL: outer nuclear layer; opl: outer plexiform layer. Tu: olfactory tubercle; VI: layer VI of the cortex; VMH: ventromedial hypothalamic nucleus; **a-b**, **c-h **are coronal sections; **c **is a sagittal section. Magnification: (**a-b**) 25×; (**c**) 5×; (**d-h**) 50×; (**i**) 100×.

**Figure 3:**
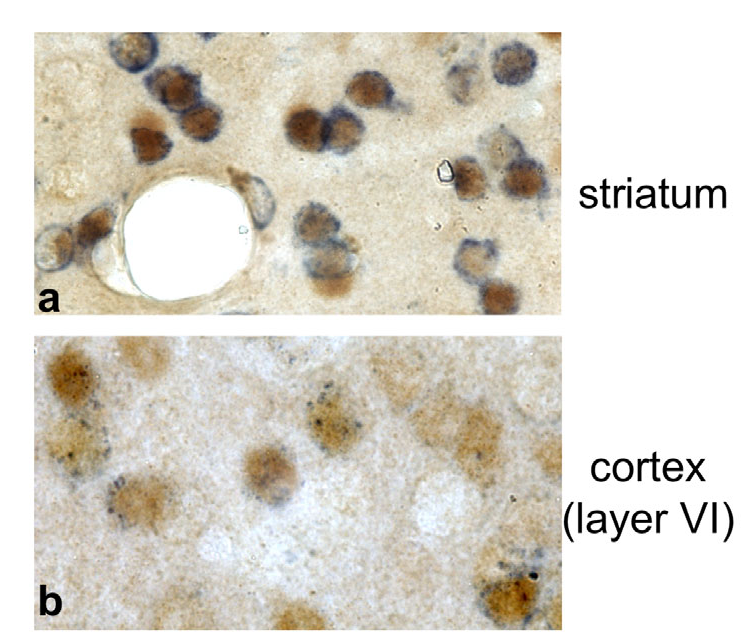
**Expression of D1Cre in neurons expressing D1R mRNA**. In situ hybridization with a riboprobe specific for D1R mRNA (blue) followed by immunohistochemistry with Cre specific antibody (brown) showing D1Cre and D1R mRNA coexpression in striatal neurons (**a**) and in the cortex (**b**). (**a**,**b**) 1000×.

The modified YAC was used to generate 5 independent transgenic lines (D1Cre lines A, R, S, T and U). Two lines (T and U) carried incomplete copies of the transgene as assessed by screening for the presence of the extremities of the YAC arms (data not shown). Examination of Cre expression by immunohistochemistry in the three remaining lines (A, R and S) revealed a virtually identical pattern of expression (Fig. 1). Moreover, the level of expression was in direct correlation with the copy number of the transgene (Fig. 1). Detailed analysis of the Cre expression pattern was conducted with line A which carries three copies of the YAC transgene. Expression was prominent in the major projections areas of the dopaminergic system, including striatum, nucleus accumbens, olfactory tubercles and prefrontal cortex (Fig. 2). Clear but lower expression was seen in layer VI of the cortex. In hippocampus, CA2 and scattered cells in the dentate gyrus expressed Cre (Fig. 2). Several thalamic nuclei and the ventromedian hypothalamic nucleus also expressed Cre (Fig. 2). In retina, robust staining was observed in the inner nuclear layer (Fig. 2). In order to verify that Cre is expressed in neurons expressing the D1R mRNA, we have performed in situ hybridization with a specific D1R riboprobe in combination with immunohistochemical detection of Cre expression in striatum and cortex (Fig. 3a, b).

**Figure 4:**
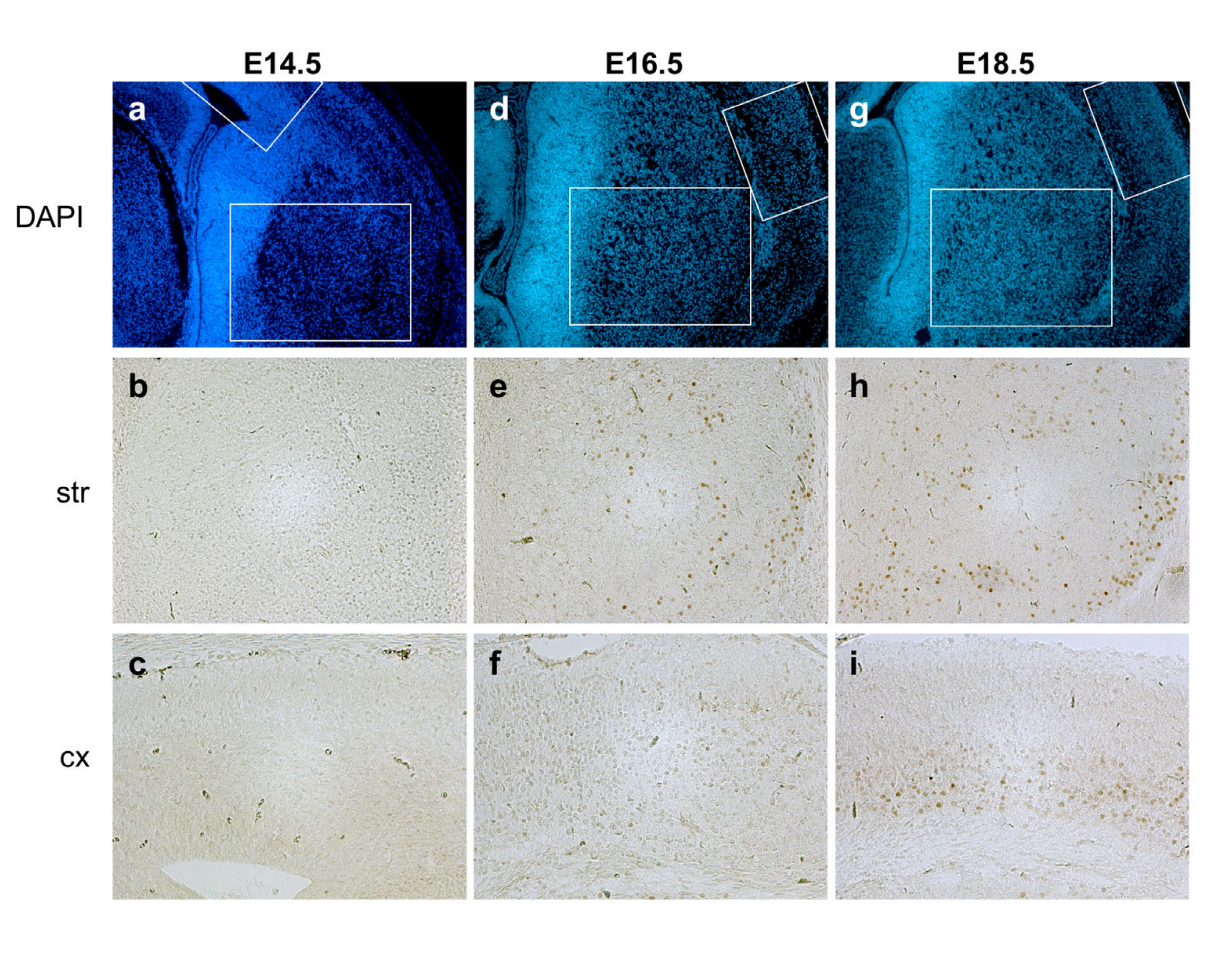
**Cre expression during embryonic development**. DAPI staining on coronal sections of embryos at E14.5, E16.5 and E18.5 (**a**,**d**,**g**). The regions analyzed in striatum and cortex by immunohistochemistry with Cre antibody are boxed. No Cre positive cells at E14.5 are visible (**b-c**). At E16.5 Cre expressing cells are detected in the striatum (str) and a few scattered weakly expressing cells are observed in the cortex (cx) (**e-f**). At E18.5, more Cre positive cells are found in the striatum. Cre expression appeared in the cortical plate, in the presumptive layer VI (**h-i**). Magnification: (**a**,**d**,**g**) 100× (**b**,**c**,**e**,**f**,**h**,**i**) 200×.

**Figure 5:**
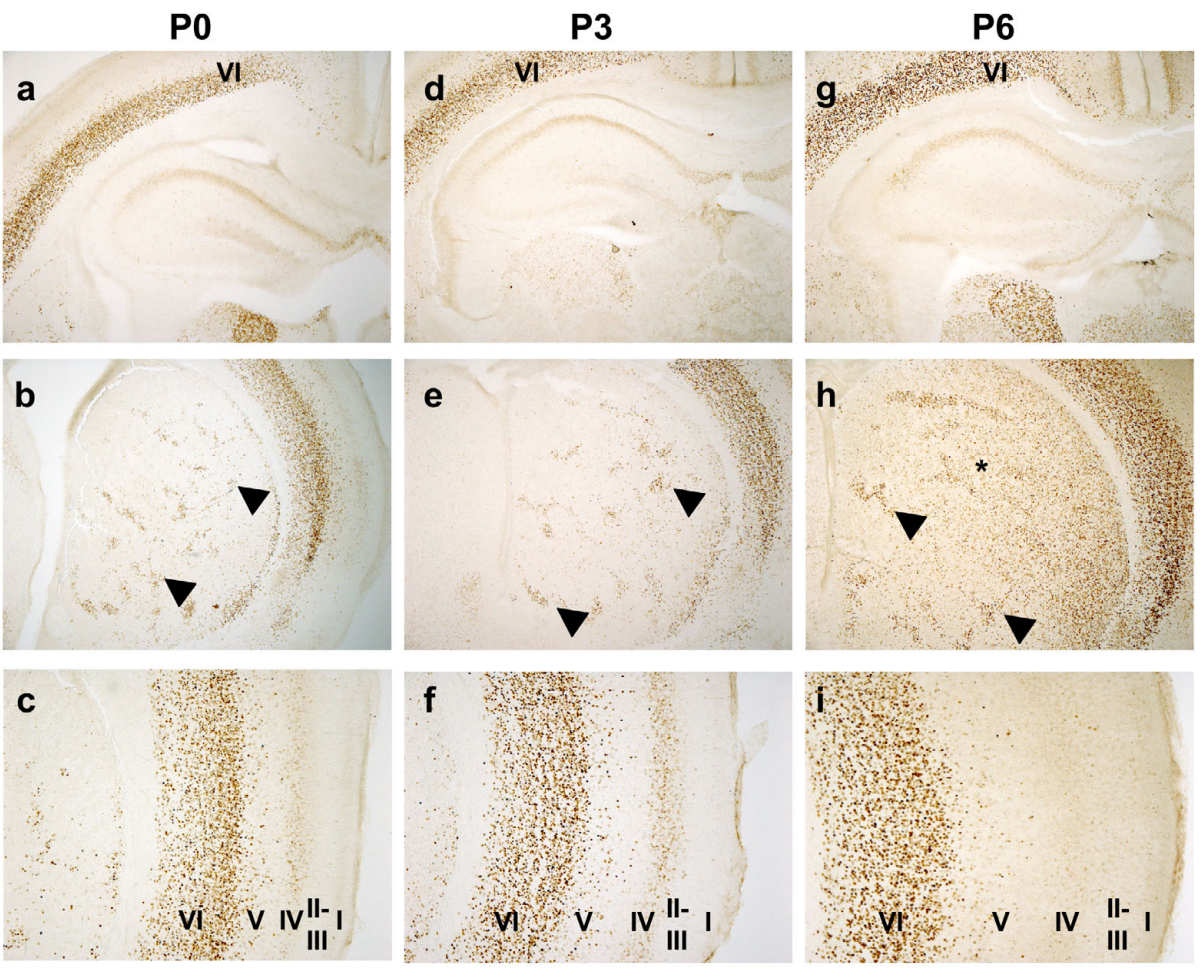
**Cre expression during postnatal development**. Immunohistochemical localization of Cre. At P0 (**a-c**) and P3 (**d-f**), striatal Cre is restricted to the patch compartment (arrowheads). Expression in layer VI of the cortex is present already at birth. Transient and very weak expression is visible in the CA1 hippocampal region (**a,d,g**) and in layer IV of the cortex (**c,f,i**). At P6 (**g-i**), striatal Cre expression starts in the matrix compartment (asterisk), between the patches (arrowheads). Expression in CA1 and cortex layer IV disappears progressively. Magnification: (**a**,**b**,**d**,**e**,**g**,**h**) 50×; (**c**,**f**,**i**) 100×.

The dynamics of Cre expression was examined during embryonic development and the postnatal period. D1R mRNA expression in the striatum has been reported at E13 in mice [14], therefore we have analyzed Cre protein at E14.5. At E14.5, Cre could not be detected in striatal anlage nor in any brain region (Fig. 4a–c and data not shown). Cre expressing cells appeared in striatal patches, the so-called striosomes at E16.5 and a only very few weakly expressing cells could be detected in the developing cortex (Fig. 4d–f). At E18.5 the number of Cre expressing cells increased in the striatum as well as in the presumptive layer VI of the cortex (Fig. 4g–i). At postnatal days 0 and 3 (P0 and P3), Cre recombinase was still predominant in the striosomal neurons and layer VI of the cortex (Fig. 5). At P6, Cre expression started to be seen between the patches. Cre expression expanded progressively until the adult stage. Transient and very weak expression was observed in the CA1 region of the hippocampus and in layer IV of the cortex (Fig. 5). These results indicate that Cre starts to be expressed, albeit at low levels, at E16 in the striosomal neurons of the striatum. At the end of the first week after birth, Cre expression is seen also in cell bodies of the matrix, recapitulating the pattern of expression described for D1R mRNA [15-17].

**Figure 6:**
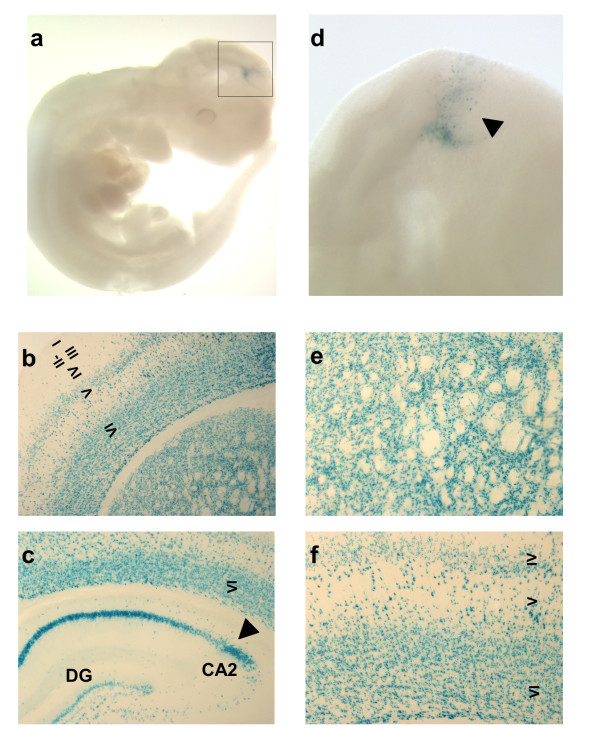
**Cre activity in embryonic and adult brain in *R26R*^D1Cre ^mice**. (**a**) Cre activity is analyzed by expression of beta-galactosidase activity (blue staining) at E10.5. The reporter mice are heterozygous for the *R26R *allele. Positive signal is present in the developing brain at this stage in the tectum above the mesencephalic fold (boxed area), as shown also in (**d, **arrowhead). In the adult brain, beta-galactosidase activity analyzed in coronal vibratome sections is present in striatum (**b,e**), cortex (**b,f**) and hippocampus (**c**). CA2: hippocampus CA2 field; DG: dentate gyrus; VI: layer VI of the cortex. Magnification: (**a**) 20×, (**d**) 40×, (**b,c**) 50×, (**e,f**) 100×.

In order to assess the specificity and the onset of Cre activity we have crossed D1Cre transgenic mice with the *R26R *reporter line [18] (Figure 6). We rule out the possibility of recombination in regions outside the CNS as shown at E11 by beta-galactosidase assay in the *R26R*^D1Cre ^reporter mice (Fig. 6a). Interestingly, we were able to detect Cre activity in the tectum above the mesencephalic region (Fig. 6d). The pattern of recombination induced by Cre recombinase was also analyzed in the adult brains (Fig. 6b, c, e, f). Beta-galactosidase activity in *R26R*^D1Cre ^was induced not only in the major areas of Cre expression shown in Figure 2, but also layers in IV to VI in the cortex (Fig. 6b, f) and in the CA1 region (Fig. 6c), expressing the Cre recombinase in early postnatal development (Figure 5).

**Figure 7:**
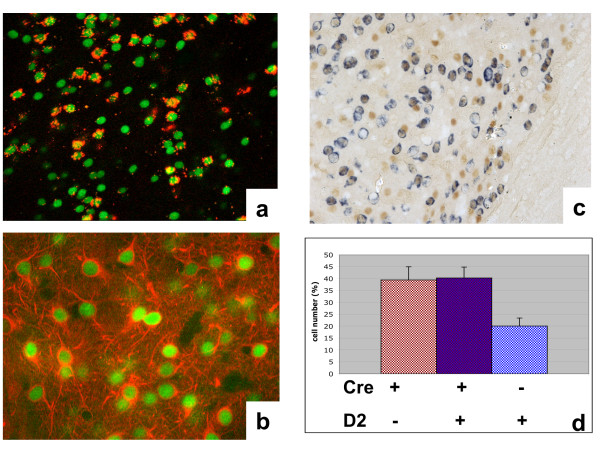
**Cre expression in striatal neurons**. (**a**) Retrograde labeling of striatonigral neurons within the striatum (red fluorescence). Cre was labeled by immunofluorescence (green fluorescence). (**b**) Double immunofluorescence against Cre (green fluorescence) and neuron-specific class III tubulin (TujI monoclonal antibody, red fluorescence). (**c**) Expression of Cre protein in neurons expressing D2R mRNA by in situ hybridization with a riboprobe specific for D2R mRNA (blue) followed by immunohistochemistry with Cre specific antibody (brown). (**d**) Quantification of Cre positive neurons expressing D2R mRNA. Neurons expressing either Cre protein or D2R mRNA were also counted. Reported is the % of neurons per each group. The data are expressed as mean ± SEM. **a-b-c **are coronal sections; (**a,b **400×; (**b**) 1000×.

We next examined whether Cre expression was confined to a specific subpopulation of striatal neurons. In the striatum, two subtypes of spiny neurons are involved in the so called "direct-" and "indirect-pathway". The direct pathway originates from striatal neurons projecting directly to the medial globus pallidus (GPm) and the substantia nigra pars reticulata (SNr). The neurons contributing to the indirect pathway are instead connected to these areas via the basal ganglia. Although it is still a matter of debate, it has been classically thought that the direct pathway neurons express the D1 dopamine receptor and the indirect-pathway neurons express the D2 receptor (D2R) [19,20]. In order to establish whether Cre expression is confined to a specific subpopulation, we retrogradely labeled neurons from the striatonigral pathway by stereotaxic injection of red fluorescent latex spheres into the substantia nigra pars reticulata and localized Cre within the striatum by immunofluorescence (Fig. 7a). Double labeling of neuron-specific class III beta-tubulin and Cre recombinase showed that the majority of striatal neurons express the Cre recombinase driven by the *Drd1a *gene (Fig. 7b). This experiment showed that Cre expression was not restricted to the retrogradely labeled neurons which correspond to neurons of the direct pathway that project directly to the substantia nigra.

To further assess the subpopulation specificity of the D1Cre transgene, we checked whether also D2R-positive cells express Cre protein in the striatum. Therefore, we performed in situ hybridization by using a D2R-specific riboprobe followed by immunohistochemistry with Cre-specific antibody (Fig. 7c). This experiment shows that D1Cre is expressed also in about 40% of D2R-expressing cells, and that only about 20% of D2R positive cells do not express Cre at detectable levels (Fig. 7d) while about 40% of the neurons express D1Cre but not D2R mRNA. Therefore, we conclude that at least 80% of the striatal neurons express D1Cre. It has to be mentioned that some studies indicate that there is only very little overlap in the number of neurons expressing both receptors [19,21]. According to these studies we were not expecting expression of D1Cre in D2R positive cells to such an extent. However, depending on the sensitivity and resolution of the adopted technical approach, others studies reveal that there is a higher degree of overlap [22] and that possibly all dopamine receptor-containing neurons, in the striatum, express both receptor types [23,24]. Our study does not solve this debate about the degree of overlap between D1R and D2R, however, the finding that Cre expression is detectable in both neuronal subpopulations, D1R- and/or D2R-expressing neurons, suggests that these receptors are at least partially coexpressed in a certain proportion of striatal neurons (about 40%).

**Figure 8:**
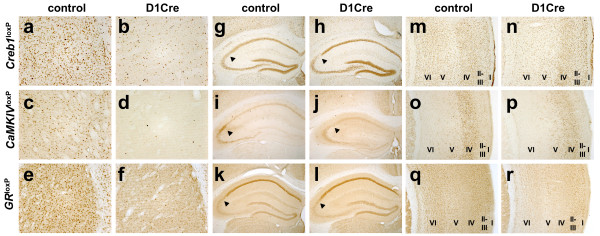
**Pattern of recombination in adult *Creb1*^D1Cre^, *CaMKIV*^D1Cre ^and *GR*^D1Cre ^mice**. Recombination is shown in striatum (**a-f**), hippocampus (**g-l**) and cortex (**m-r**). Recombination is followed by disappearance of CREB (**a,b,g,h,m,n**), CaMKIV (**c,d,i,j,o,p**) and GR proteins (**e,f,k,l,q,r**) in the respective mutants. All animals are homozygous for the respective loxP-modified allele in the case of *Creb1*^loxP^, *CaMKIV*^loxP ^and *GR*^loxP ^mice. Control animals are devoid of the D1Cre transgene while mutant animals carry the D1Cre trangene, as indicated. The hippocampus CA2 field is indicated by arrowheads. The cortical layers are indicated. Magnification: (**a**-**f**) 200×; (**g**-**l**) 50×; (**m**-**r**) 54×.

The expression of D1Cre not only in D1R positive neurons (Fig. 3a), but also in D2R positive cells (Fig. 7d), makes the D1Cre line suitable for gene inactivation in virtually all dopaminoceptive neurons of the striatum. The pattern of recombination induced by Cre recombinase was verified by crossing D1Cre transgenic animals to 3 loxP lines, *Creb1*^loxP ^[25], *CaMKIV*^loxP ^[26], *GR*^loxP ^[18,27]. Recombination was visualized by loss of CREB, CaMKIV and GR proteins in the respective mutants. Loss of CREB, CaMKIV and GR proteins was extensive (at least 90%) in the main regions of Cre expression, striatum, nucleus accumbens, olfactory tubercles, and layer VI of the cortex (Figure 8). In the hippocampus, expression of Cre in the CA2 subfield results in loss of CREB, and CaMKIV in the corresponding cells. No or very marginal loss of CREB, CaMKIV and GR was observed in CA1 and layer IV of the cortex, in spite of the transient expression of Cre during the early postnatal period. These results underscore the importance of verifying recombination in the context of the loxP modified allele of interest.

## Discussion

The pattern of expression obtained with the D1Cre YAC transgene was very reproducible between transgenic lines. The expression level was essentially only dependent on the copy number of the transgene. These observations illustrate well the properties of transgenes based on large DNA segments: position-independent and copy-number dependent expression of the transgene. This correlation between expression pattern and copy number usually does not apply to plasmid-based constructs [28]. The pattern of Cre expression as seen in the D1Cre transgenic animals is very close to the pattern of expression of the endogenous *Drd1a *gene reported in previous *in situ *hybridization studies [29]. Moreover, the developmental and postnatal patterns of expression match the patterns described for the endogenous *Drd1a *gene [15-17]. We have also performed in situ hybridization with D1R riboprobe in combination with Cre immunohistochemistry in adult cortex and striatum and found high levels of overlap (Fig. 3a, b).

Several publications have suggested that D1 and D2 receptors are segregated specifically on distinct neuronal populations of the rat striatum that projects directly to substantia nigra pars reticulata and to globus pallidus respectively [20,30]. Some other publications have however demonstrated that D1 and D2 might actually be coexpressed [22-24]. These discordant studies clearly indicate that a fine modulation of expression levels of D1R and D2R takes place in the dopaminoceptive neurons. In our transgenic line, we observe that Cre is expressed in a majority of striatal neurons, but it remains open whether our vector, even though it contains large segments of 5'- and 3'- flanking sequences, carries all necessary elements required to achieve this fine modulation of expression levels within striatum.

A potential application of D1Cre transgene for isolation of mutated dopaminoceptive cells is represented by the generation of double transgenic mice with BAC-D1GFP and BAC-D2GFP obtained by the GENSAT BAC Transgenic project [31]. The D1Cre expression pattern matches quite closely the BAC-D1GFP pattern in the medium spiny neurons of the striatum, therefore one would expect to isolate recombined GFP positive cells. However deeper analysis by costaining is required to compare the two transgenic lines.

When recombination was verified in the context of three loxP-modified alleles, loss of the respective proteins were largely confined to the main areas of Cre expression. Thus loss of CREB, CaMKIV and GR were extensive in striatum, layer VI of the cortex and CA2 of the hippocampus (Figure 8). However, induction of beta-galactosidase activity in *R26R*^D1Cre ^reporter mice showed a more widespread pattern of recombination in layer IV of the cortex and in the CA1 of the hippocampus (Figure 6). One possible explanation for this could be that scattered recombination, as caused by weak and transient Cre expression occurring at early postnatal stages (Figure 5), is easily underestimated when followed by loss of the respective protein but very visible when revealed by beta-galactosidase staining. This may be also related to a tissue-specific difference in the stability of CREB, CaMKIV and GR proteins in CA1 and layer IV.

In view of the patterns of recombination restricted to the major dopaminoceptive regions as seen in the context of the CREB, CaMKIV and GR mutations, the D1Cre line should be useful to evaluate the contribution of these, and others, signaling molecules to the dopaminergic system. The D1Cre line, combined with specific pharmacological treatment may provide a powerful tool do genetically dissect signalling pathways underlying dopaminergic neurotransmission.

## Conclusion

We present the generation and characterization of a transgenic mouse line expressing the Cre recombinase under the control of the D1R regulatory elements. We show that this transgenic line can be used successfully for the conditional ablation of specific genes important for biological processes involving dopamine signaling.

## Methods

### D1Cre YAC construction

The small insert Princeton mouse YAC library [12] was screened by PCR (primers: 5'-TTT CAT CCT CCC TCA TAA GC-3' and 5'-TTCGACAGGGTTTCCATTAC-3'). To allow for subsequent modification, the YAC was transferred into the yeast strain YPH925 [33]. To insert the Cre recombinase into the *Drd1a *gene, a targeting vector was constructed by cloning a 800 bp 5' homology region (primers:5'-GGG GCG GCC GCG GTC CTG CCC TAA GAA CGA G-3'and 5'-ACC AAG CTT AGC CAG ACT TCC CCC-3'), the nlsCre open reading frame (*Hind*III-*Eco*RI fragment from pHD2 nlsCre, [13]) and a 500 bp 3' homology region (primers: 5'-TTT GGG TGG GCG AAT TCT TCC CTG AAC CCC ATT ATT TAT-3' and 5'-GAT AAT ACT CCC AAA CTG GAT TTC AGA GCC GAA GTC ATT T-3') bearing a unique *Xba*I restirction site into the *Not*I and *Bam*HI sites of the pRS306 vector [34]. The *Xba*I linearized targeting vector was then used to modify the *Drd1a *containing YAC by classical "pop-in pop-out" modification [35].

### Generation of transgenic animals

The YAC DNA from one of the positive colonies was purified as described [36] and microinjected into the pronucleus of FVB/N oocytes. Founders were identified by Southern blot analysis of *Bam*HI digested genomic DNA using the 5' homology region as a probe. Since this probe hybridizes identically to the endogenous *Drd1a *gene and to the modified YAC, it was used to determine the copy number of the transgene in the various founders. For subsequent routine genotyping, dot blot analysis was performed with a probe for the Cre open reading frame. The transgenic line was maintained by backcrossing to C57Bl/6. To generate the respective mutant animals, the D1Cre transgenic mice were first crossed to mice homozygous for the respective loxP-modified allele. Offspring hemizygous for the transgene and heterozygous for the loxP-modified allele were in turn crossed to mice homozygous for the loxP-modified allele to generate mutant animals with an expected frequency of 25%. We did not observe cases of germline recombination with this transgenic line.

### Histology

Paraffin sections (7 μm) from mouse embryos at different developmental stages and vibratome sections (50 μm) prepared from brains of perfused mice (0.1 M phosphate-buffered 4% paraformaldehyde) were used for immunohistochemical labeling. The following antibodies were used: a rabbit polyclonal anti-Cre antibody (1:3,000, [37]), a rabbit polyclonal anti-CREB antibody (1:3000), a goat polyclonal anti-CaMKIV antibody (1:200, Santa Cruz, cat. Sc-1546), a rabbit polyclonal GR antibody (1:2000, Santa Cruz, cat. Sc-1004), a mouse monoclonal anti class III beta-tubulin antibody (1:400, clone TujI, Covance, cat. MMS-435P). Immunohistochemistry was performed using the avidin-biotin system (Vectastain, Vector Labs). Immunofluorescence was achieved using Alexa488 coupled anti-rabbit antibody and an Alexa594-coupled anti-mouse antibody (Molecular Probes) as secondary antibodies. Beta-galactosidase activity was revealed by incubating embryos or vibratome sections at 37°C overnight in X-Gal staining solution (5 mM potassium hexacyanoferrate (III), 5 mM potassium hexacyanoferrate (II), 2 mM MgCl_2_, 0.01% NP-40, 0.02% sodium deoxycholate, 1 mg/ml X-gal, 20 mM Tris/HCl, pH7.5).

Non-radioactive in situ hybridization was performed on vibratome sections (20 μm) prehybridized, after a step with Proteinase K, at 70°C for 1 hour. Hybridization with specific riboprobe occurs overnight at 70°C in the hybridization solution. After two washing steps the sections were incubated with the anti-digoxygenin antibody (1:10000) in 20% normal swine serum. The development of the reaction was done with NBT/BCIP (Roche). After washing in PBS and PBST, the sections were processed for immunohistochemistry as described above.

### Cell counting

To estimate the % of neurons expressing D2R and Cre, cells positive for both signals were counted in nine nonoverlapping microscopic field at 400× magnification. Neurons expressing either Cre protein or D2R mRNA were also counted. The data are expressed as mean ± SEM.

### Retrograde tracing of striatonigral neurons

Mice were injected stereotaxically into the right and left substantia nigra pars reticulata (AP = -3.28 mm, DV = -4.15 mm, L = -1.3 mm and +1.3 mm) with 2 μl of a carboxylate-modified red latex FluoroSpheres solution (Molecular Probes, cat. L-2783,). Three days later, the animals were perfused through the ascending aorta with 0.1 M phosphate-buffered 4% paraformaldehyde and the brains were removed to be postfixed for 4 hr at 4°C. Cre immunofluorescence was performed on cryostat-cut free-floating 30 μm-thick sections. Sections were examined on a confocal microscope (MRC 1024, Bio-Rad Laboratories, Hemel Hempstead, Hertfordshire, U.K.) fitted on an inverted microscope (Axiovert 100, Zeiss, Oberkochen, Germany).

## List of abbreviations

YAC: yeast artificial chromosome; CREB: cAMP-responsive element binding protein; CaMKIV: Ca^2+^/Calmodulin-dependent protein kinase IV; GR: glucocorticoid receptor; D1R: dopamine D1 receptor; D2R: dopamine D2 receptor

## Authors' contributions

TL conceived the study, generated the D1Cre transgenic line and most of the conditional mutants used for recombination analysis, drafted the manuscript. RP carried out part of the histological characterization of the line, participated in the coordination of the study and helped to draft the manuscript. DD performed the retrograde labeling experiment. MW performed the in situ hybridization experiments. EC generated the CamKIV floxed mice. MT and FT generated the GR^D1Cre^mutants used for the recombination analysis. SNS conceived and participated in the retrograde labeling experiments. GS participated in the design and coordination of the study and in the draft of the manuscript. All authors read and approved the final manuscript.
